# The Food Adulteration Law

**Published:** 1881-08

**Authors:** 


PASSAGE OF THE FOOD ADULTERATION LAW.—We publish elsewhere the text of the law to prevent the adulteration of food and drugs, and cheerfully extend our congratulations to the editor of the *Sanitary Engineer*, and also the appropriate editorial of our confrere upon his successful efforts in behalf of this important measure. The subject should interest all very deeply, as it concerns not only our health but our lives also.

